# Food cue reactivity: Neurobiological and behavioral underpinnings

**DOI:** 10.1007/s11154-022-09724-x

**Published:** 2022-04-28

**Authors:** Scott E. Kanoski, Kerri N. Boutelle

**Affiliations:** 1 Department of Biological Sciences, Human and Evolutionary Biology Section, University of Southern California, Los Angeles, CA USA; 2 Department of Pediatrics, Herbert Wertheim School of Public Health and Human Longevity Science, and Psychiatry, University of California San Diego, San Diego, CA USA

**Keywords:** Obesity, Overeating, Pavlovian, Food cue, Conditioning

## Abstract

The modern obesogenic environment contains an abundance of food cues (e.g., sight, smell of food) as well cues that are associated with food through learning and memory processes. Food cue exposure can lead to food seeking and excessive consumption in otherwise food-sated individuals, and a high level of food cue responsivity is a risk factor for overweight and obesity. Similar food cue responses are observed in experimental rodent models, and these models are therefore useful for mechanistically identifying the neural circuits mediating food cue responsivity. This review draws from both experimental rodent models and human data to characterize the behavioral and biological processes through which food-associated stimuli contribute to overeating and weight gain. Two rodent models are emphasized – cue-potentiated feeding and Pavlovian-instrumental transfer – that provide insight in the neural circuits and peptide systems underlying food cue responsivity. Data from humans are highlighted that reveal physiological, psychological, and neural mechanisms that connect food cue responsivity with overeating and weight gain. The collective literature identifies connections between heightened food cue responsivity and obesity in both rodents and humans, and identifies underlying brain regions (nucleus accumbens, amygdala, orbitofrontal cortex, hippocampus) and endocrine systems (ghrelin) that regulate food cue responsivity in both species. These species similarities are encouraging for the possibility of mechanistic rodent model research and further human research leading to novel treatments for excessive food cue responsivity in humans.

Obesity is a significant public health concern, as more than 70% of American adults [1, 2] and over 40% of children [3] have overweight or obesity. Obesity-related conditions (i.e., stroke, hypertension, diabetes, heart disease) are some of the leading causes of preventable death, and overconsumption of calorically dense foods is one of the most proximal causes of the elevated overweight and obesity rates [4].

Today’s environment encourages excess energy intake and discourages energy expenditure [5–8] and has been implicated as one of the drivers of the obesity epidemic [9, 10]. An individual’s level of food cue responsivity (FCR) is a result of genetic risk factors interacting with the environment, through learning, neural changes, and memory [11]. Food cues include visual, auditory, olfactory, emotions, situations and any other cues (e.g., time) that are associated with food-related memories [4]. Specifically, FCR is defined as responses to these cues that ultimately drive overeating and weight gain [12]. Responses to food cues include psychological responses (e.g., craving, urge), physiological changes (salivation, hormone secretion), and neurocognitive responses (brain activation and allocation of attentional resources) [13]. Thus, it is important to understand the psychological, behavioral, and neurobiological mechanisms that underly FCR.

Beyond genetic susceptibility, overeating develops through basic learning processes, including Pavlovian and operant conditioning [14, 15]. In today’s food environment, there are multiple opportunities to associate cues in the environment with food and overeating. Through Pavlovian conditioning, these food cues become directly associated with food intake and can elicit arousal, urges to eat, cravings, expectancies, thoughts, drives and motivations to eat [16]. Operant conditioning also occurs, where the association of food seeking actions or eating are paired with the reinforcing effects of eating [17]. These two learning processes act in concert [18] and the presentation of Pavlovian food cues can increase operant responding for palatable food (e.g., Pavlovian-instrumental transfer, described in more detail below) [19, 20]. Food cues can also acquire secondary reinforcing properties through their association with food-directed actions [21] and can eventually elicit the operant behavior [22–24]. Food cues that are present when operant actions are reinforced can influence operant responding by “setting the occasion” for the action–outcome relationship rather than eliciting or motivating behavior through their simple direct association with food [25]. Once established, FCR also provides opportunities for higher-order cognitive processes to take place, including planning to consume food in the future [26]. Additionally, food cues can grab attention resulting in a bias in attentional resources for food cues (attentional bias), which is shown to be associated with FCR [27, 28]. This increased attention to food cues may provide more opportunities for both basic and complex learning processes to take place, thereby perpetually increasing the strength of FCR.

A primary goal of this review is to draw from preclinical work to understand neuronal circuit-level mechanisms driving two key behavioral phenomena that specifically relate to FCR, cue-potentiated feeding and Pavlovian-instrumental transfer. Next, we review the human data on FCR, overeating and weight gain. Finally, we conclude with recommendations for future research based on gaps in the literature.

## Insights from preclinical models

Preclinical animal models have proven to be invaluable for gaining mechanistic understanding of the neurobiological controls of food intake and energy balance. In this section we describe two rodent models, cue-potentiated feeding (CPF) and Pavlovian-instrumental transfer (PIT), and review literature derived from these models that contribute to the current understanding of neurobiological systems that regulate stimulus-driven food seeking and consumption. We note that while various other rodent appetitive paradigms provide additional mechanistic insight into stimulus-induced eating (e.g., sign- and goal-tracking, incentive learning, US devaluation; see [22, 29] for review on these topics), our focus is on CPF and PIT as these procedures provide a direct window into the capacity of food-associated cues to promote excessive food seeking and/or consumption. Moreover, we emphasize these models as their underlying neural substrates have been systematically investigated for decades, thus offering a rich literature to draw from.

### Cue-potentiated feeding

#### Neural pathways

**Fig. 1:**
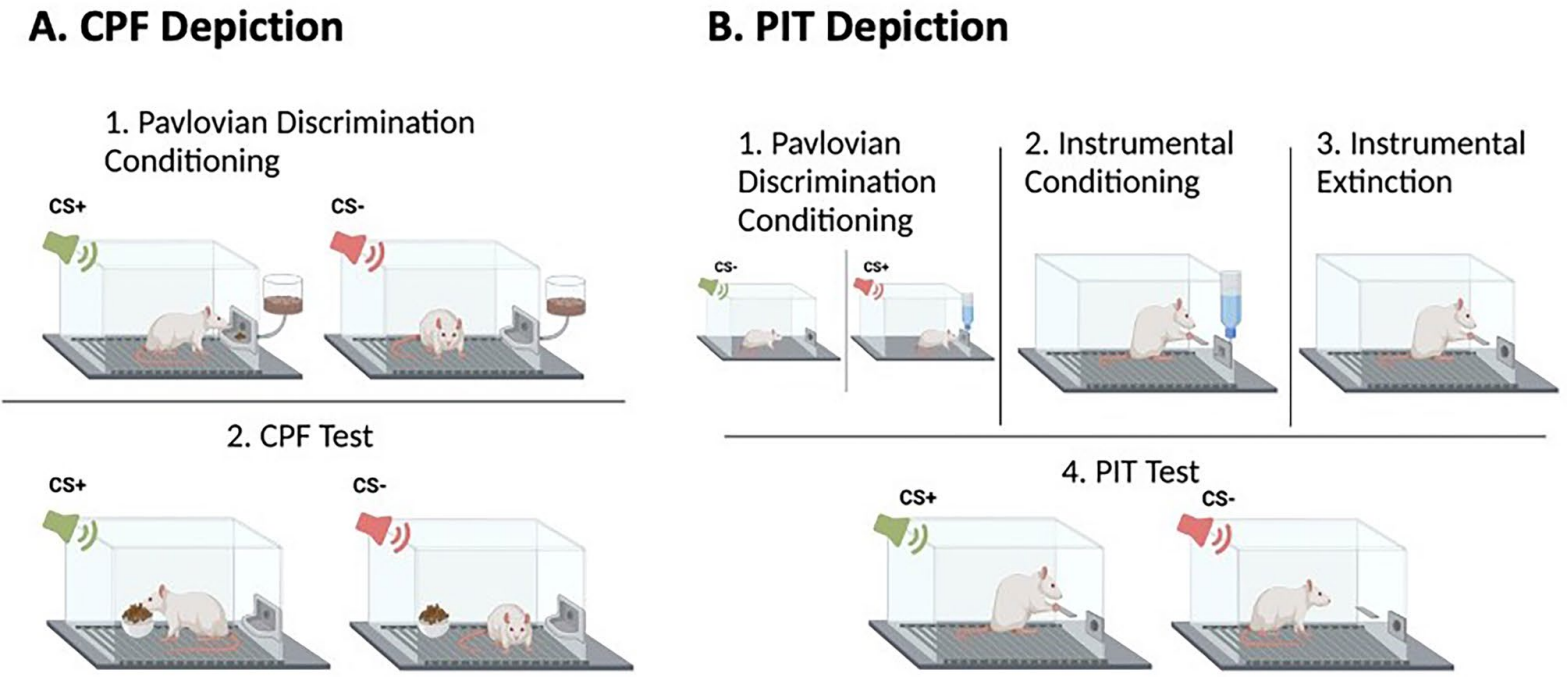
Behavioral procedures for rodent models of CPF and PIT

FCR, in pre-clinical models, is commonly referred to as “cue-potentiated feeding” (CPF) or “stimulus-induced eating” and is based on associative learning mechanisms through which external cues that have previously been paired with access to and consumption of highly palatable food gain stimulus control over behavior [30, 31]. These models involve a training phase, typically conducted under conditions of food restriction to facilitate conditioning, in which the presentation of discrete cues (e.g., light, tone; CS +) reliably predicts the delivery of palatable food (the US) and the presentation of a control stimulus is not associated with food delivery (CS-). During a test session, food-sated animals are typically given free access to the US while being exposed to various CS + and/or CS- presentations. Evidence for CPF is based on increased consumption during (or after) CS + presentations compared to either comparable CS- presentations or a no stimulus condition (Fig. 1A). Studies have demonstrated that contextual cues can also function as a CS + and stimulate consumption in sated rats without any discrete cues present [32–34]. Evidence suggests that CS + exposure in rodent CPF models does not induce a general state of hunger, but rather, is selective to the specific food/US used during training [35], although this specificity, at least for contextual-based CPF, can be overcome when a variety of foods are used as USs [34]. Thus, CPF in rodents can be considered a direct analog to FCR.

CPF research has used a combination of bilateral neurotoxic lesions, lesion-based disconnection between brain regions (unilateral and contralateral lesions of two brain regions with exclusively ipsilateral connections), behavioral, neural tract tracing, and immediate early gene mapping approaches to identify brain regions and connections that are necessary for CPF in rats. Results show that lesions to the basolateral amygdala (BLA), but not the amygdala central nucleus (CeA), eliminated the CPF effect to discrete food-conditioned cues [36]. Furthermore, a disconnection between the BLA and the lateral hypothalamic area (LHA), while having no effect on baseline eating or body weight gain, blocked the discrete cue CPF effect observed in control animals [37]. Presumably this outcome is based on ablation of a BLA to LHA pathway, although possibility of an LHA to BLA pathway cannot be ruled out given that this approach completely eliminates communication between the two brain regions. Additionally, the medial prefrontal cortex (mPFC) is a critical brain region mediating the capacity of contextual food cues to trigger excessive eating, as bilateral mPFC lesions eliminated CPF induced by contextual cues associated with food reward [32].

#### Neuropeptides

More recent work has extended these findings and provides a more complete neural circuit-level understanding of CPF control, including connections to hypothalamic neuropeptide systems. Using a systemic administration of an antagonist for the receptor for orexin (aka, hypocretin), a neuropeptide produced in the LHA, reduced discrete cue-induced CPF in rats yet had no effect on baseline food intake [38]. Further, the orexin receptor antagonist treatment increased food cue-induced c-Fos induction (a marker of neuronal activation) in the mPFC and in the paraventricular nucleus of the thalamus (PVT). A role for mPFC orexin signaling in CPF is further supported by their subsequent work revealing that mPFC-LHA disconnection reduced CPF induced by discrete food cues without influencing food-cue learning, and that CPF was also blocked with mPFC-specific orexin receptor blockade [39]. These findings collectively support that the mPFC is functionally associated with CPF for both discrete and contextual food cues, and that the neuropeptide orexin is an important neurochemical signal for CPF.

Like orexin, melanin-concentrating hormone (MCH) is an orexigenic neuropeptide produced predominantly within the LHA [although in different neurons than orexin] [40]. Genetic deletion of MCH in mice significantly impairs discrete cue-induced CPF expression in food-sated mice [41]. This suggests that two distinct LHA-derived neuropeptide systems, orexin and MCH, are involved in cue-potentiated feeding, thus providing neurochemical specificity to early work identifying a role for the LHA in CPF.

#### Peripheral signals

In addition to the LHA-derived neuropeptides discussed above, emerging evidence suggests that the stomach-derived orexigenic hormone, ghrelin, is critical in the induction of CPF. Circulating levels of ghrelin are largely determined by levels of energy restriction, with higher levels observed following a fast. However, ghrelin is also released from the stomach as an anticipatory feeding signal in response to conditioned circadian cues [42], and potentially in response to visual and other discrete food cues [43, 44]. Evidence for ghrelin’s role in CPF comes from data in mice where genetic deletion of the ghrelin receptor (GHSR1a) inhibits the capacity of discrete conditioned food cues to stimulate CPF [45]. Similarly in rats, systemic administration of a GHSR1a antagonist also blocks CPF effects in response to discrete cues [46]. The ventral hippocampus (field CA1; HPCv) is a likely candidate brain region mediating these effects as pharmacological HPCv GHSR1a activation enhances CPF relative to vehicle/control treatment [47]. Ghrelin’s role in CPF may be stimulated by the capacity of palatable food-associated cues to trigger the physiological release of ghrelin, as recent findings show that olfactory detection of a familiar, palatable food caused both an increase in active ghrelin release and a persistent overconsumption of chow [48].

#### Summary

In summary, these findings identify the LHA, BLA, PVT, mPFC, and HPCv as brain regions of importance in the mediation of CPF. Interestingly, the HPCv has monosynaptic projections to all of these other regions associated with CPF control [49]. While HPCv (field CA1) projections to LHA [50] and mPFC [51] have been identified as relevant to feeding behavior, the function of these connections with regards to CPF remains to be explored. Given that palatable food-associated olfactory cues stimulate ghrelin release [48], that HPCv GHSR1a-to-LHA signaling functionally targets LHA orexin neurons to enhance eating [52], and that both mPFC orexin receptor signaling and LHA-mPFC signaling are necessary for CPF [39], a putative model emerges in which exposure to cues associated with palatable food stimulates peripheral ghrelin release, which crosses the blood–brain-barrier to engage a [HPCv GHSR1a]-to-[LHA orexin neurons]-to-[mPFC] pathway to promote CPF. More research is required to understand the neural pathways through which MCH mediates CPF [53, 54].

### Pavlovian-instrumental transfer

Animals and humans must be able to flexibly obtain desired outcomes while also avoiding aversive outcomes. Critical to these fundamental complementary behavioral drives is the ability to learn contingent relationships between actions and outcomes via a process known as instrumental conditioning (aka, operant conditioning). In addition to action–outcome learning, Pavlovian conditioning, including the type of stimulus-outcome (CS-US) training described above for CPF procedures, can also have a powerful influence over instrumental response performance. A classic example of this in rodent models is the Pavlovian-instrumental transfer (PIT) set of procedures [20]. This behavioral paradigm typically involves an initial Pavlovian training stage in which a stimulus/CS (e.g., light, tone, or multiple stimuli) is paired with an outcome/US, which for the focus of this review is palatable food. In the next stage, one or more instrumental actions (e.g., lever press, nose poke) are trained to yield the same US (or a different US) used in the Pavlovian training stage, but absent any Pavlovian stimuli. In the final stage, a PIT test is performed where the instrumental action(s) is available, and the Pavlovian-trained stimulus/stimuli are presented periodically such that their influence on instrumental actions can be assessed. This test usually occurs following extinction of the instrumental response, and under extinction conditions, such that no US is present during PIT testing regardless of the instrumental responses made or the stimuli presented. Evidence for PIT, for example, would be a reinvigoration of an extinguished instrumental response upon presentation(s) of the CS (Fig. 1B). PIT in rodent models demonstrates food-seeking behavior that occurs after exposure to omnipresent palatable food-associated cues. Indeed, the translational relevance of PIT is strongly supported by recent findings showing that selectively-bred obesity-prone rats show heightened PIT (w/ palatable food as US) relative to obesity-resistant rats [55], and that PIT magnitude in outbred rats is positively associated with susceptibility to diet-induced obesity [56].

PIT procedures can be dissociated into two different subcategories that, as described in more detail below, appear to differ with regards to the underlying neurobiological substrates. “US-specific PIT” can be evaluated by comparing the effects of a Pavlovian CS on two distinct instrumental responses; one that shares the US with the CS, and another that does not. Alternatively, US-specific PIT can also be assessed with two CS + s associated with two different USs, and two distinct instrumental responses (e.g., lever press, chain pull) each yielding one of the USs used in Pavlovian training. “General PIT”, in contrast, is when stimulus control of instrumental behavior is triggered by the general motivational properties shared by the Pavlovian and instrumental training, as evidenced by a PIT effect (CS presentation enhances instrumental responding) when the Pavlovian and instrumental training phases are conducted with distinct USs (e.g., sucrose or high-fat pellets). While changes in energy status do not appear to substantially enhance or disrupt US-specific PIT, General PIT is enhanced or reduced with energy restriction or satiation, respectively, prior to testing [57].

#### Mesostriatal control

The ACB is critical for PIT, as lesions to the nucleus accumbens shell (ACBsh) but not core (ACBc) impairs US-specific PIT [58]. In subsequent work complementing the lesion approach with pharmacological inactivation of the ACB subregions (via targeted muscimol infusions), data shows that ACBsh is required for the expression of US-specific, but not General PIT, whereas the opposite is true for the ACBc [59]. These findings collectively indicate that the ACBc mediates the general excitatory effects of food-associated cues, whereas the ACBsh mediates outcome-specific reward predictions on instrumental performance.

Recent studies identify a role for glutamate, dopamine, and acetylcholine signaling in the ACB in mediating PIT. For example, in studies using a Single US PIT design, the PIT effect is blocked by ACBc administration of an glutamatergic AMPA receptor antagonist [55], a dopamine 1/2 receptor antagonist [60], or a cholinergic muscarinic receptor antagonist [61]. Interestingly, ACBc blockade of cholinergic nicotinic receptors augmented PIT [61], suggesting a complex bidirectional modulation of cue-driven food seeking behavior by ACB acetylcholine signaling. A functional role for ACBc dopamine signaling in mediating PIT is further supported by data showing that the magnitude of food cue-evoked dopamine release in the ACBc (using fast-scan cyclic voltammetry) correlated with the magnitude of US-specific PIT behavioral effect [62]. There is an intriguing yet incompletely understood interaction between ACBc acetylcholine and dopamine signaling in mediating PIT, as blockade of ACBc muscarinic receptors not only reduced PIT (as indicated above), but also suppressed the ACBc cue-evoked DA response.

Emerging findings indicate that the source of dopaminergic input to ACBc mediating PIT comes from the midbrain ventral tegmental area (VTA). For example, inactivation of the VTA disrupts Single US PIT [63]. Subsequent work using a PIT design that distinguished between US-specific and General PIT revealed that VTA inactivation attenuated these two PIT effects equally [57]. A specific role for VTA dopamine signaling in mediating these effects comes from findings showing that chemogenetic inhibition of VTA dopamine neurons blocks Single US PIT, likely through downstream ACB signaling as the same study showed similar results following chemogenetic inhibition of VTA-originating dopaminergic inputs to the ACBc but not the mPFC [64]. This pathway likely involves dopamine 1 (D1R), and not 2 receptor (D2R) signaling in the ACB, as D1R, but not D2R pharmacological blockade in the ACBsh abolished US-specific PIT without influencing General PIT [65]. Interestingly, in the same study blockade of either D1R or D2R in the ACBc had no effect on either US-specific or General PIT. While these results are consistent with the lesion studies described above, they are not consistent with results showing that blockade of D1R + D2R in the ACBc reduced Single US PIT [60], although the former study blocked either D1R or D2R and the latter blocked both receptors, which may explain the discrepancy.

Recent work supports a model in which ventral pallidum (VP) to mediodorsal thalamus (MD) signaling acts downstream of VTA dopamine—> ACB signaling to mediate PIT. For example, the VP is a major downstream target of the ACB, [66] and either pharmacological inactivation of the VP or lesion-based disconnection of the VP and ACBsh blocked US-specific PIT [67]. Further, the MD receives substantial input from the VP [68], and either MD lesions [69] or lesion-based VP-MD disconnection [70] blocked the US-specificity of PIT. More research is needed to determine whether the VP- > MD mediation of PIT involves downstream signaling from VTA- > ACB signaling, as hypothesized [71], vs. functioning as a separate parallel neural network.

#### Cortical and limbic control

Similar to the CPF results discussed, the amygdala appears to also play a key role in PIT when palatable food is used as reinforcement. There was some controversy, however in early reports examining the influence of different amygdala subregions on PIT, with some studies showing BLA involvement [72, 73], and others showing CeA but no BLA involvement [74, 75]. These differences are likely based on differential PIT procedures between the studies, an issue that was at least partially resolved in a study that shows that BLA lesions abolished the US-specific but spared General PIT [76]. In contrast, CeA lesions abolished General but not US-specific PIT, suggesting that the BLA mediates palatable food outcome-specific incentive processes, whereas CeA is involved in controlling general motivational influence of food reward-related events.

Another study identified the lateral orbitofrontal cortex (lOFC) as a downstream target of BLA mediation of US-specific PIT, as chemogenetic-mediated inactivation of BLA terminals in the OFC blocked US-specific PIT, whereas inactivating the reverse pathway (OFC- > BLA) had no effect [77]. Interestingly, however, subsequent work revealed that the lOFC and mOFC inputs to BLA involve distinct connections, and that while lOFC- > BLA signaling does not appear to influence PIT, US-specific PIT is indeed mediated by mOFC- > BLA signaling [78]. Additional support for a role for the OFC in PIT comes from electrophysiological recordings from OFC neurons in awake behaving rats, where it was found that the neural representation of PIT correlated with the strength of the PIT behavioral effect [79].

#### Future directions

Similar to CPF, ghrelin signaling appears to influence PIT, although in the opposite direction. While ghrelin signaling enhances CPF in both mice and rats, peripheral administration of a ghrelin receptor antagonist in rats enhanced Single US PIT [46]. While more research is needed to understand the underlying neural substrates mediating these effects, the VTA is unlikely to be involved as, while VTA administration of ghrelin increased motivated lever pressing for palatable food under a progressive ratio schedule, it had no effect on Single US-PIT [80].

### Conclusions

The ACB and amygdala are key centers for palatable food-based PIT mediation, with the ACBsh and BLA being more linked with US-specific PIT, and the ACBc and CeA being tied to General PIT. Key upstream neural targets of these regions include the VTA dopamine neurons for modulation of ACB contributions to PIT, and the mOFC for the BLA contributions to PIT. Likely downstream targets include VP- > MD signaling from the ACB, and lOFC signaling from the BLA. Evidence for peptide system contributions to PIT thus far are predominantly from research targeting the ACB, with glutamatergic, cholinergic (bidirectionally), and dopamine signaling being functionally linked with PIT mediation. Ghrelin signaling appears to have a surprising influence on PIT, as blockade of this orexigenic system increases PIT, an outcome opposite to that predicted from CPF literature. More research is needed to understand the neural loci mediating ghrelin’s influence on PIT, as well the neural circuit-level mechanisms through which midbrain basal ganglia pathways (VTA- > ACB, VP- > MD signaling) interact and converge with telencephalic pathways (amygdala-cortical interactions) to modulate FCR.

Research on the control of feeding behavior and energy balance has largely focused on peripherally-derived hormone systems that are modulated by energy status and function to potently regulate metabolism, food intake control, and energy expenditure. Such systems include: leptin, ghrelin, cholecystokinin, glucagon-like peptide-1, amylin, and insulin. Aside from ghrelin, the contribution of these systems to palatable food cue responsivity in preclinical animal models is poorly understood. Moreover, in addition to orexin and MCH, a number of hypothalamic-derived neuropeptides potently regulate energy balance, including agouti-related peptide, pro-opiomelanocortin, neuropeptide Y, cocaine-and-amphetamine-regulated transcript, and oxytocin. While central oxytocin signaling was recently shown to not influence Single US PIT [81], its role in CPF has not been systematically investigated. Moreover, to our knowledge the role of these hypothalamic neuropeptide systems in mediating PIT is unknown.

## Insights from human studies

In humans, a variety of measures exist to assess FCR, including self-reported cravings, questionnaires, tasks, physiological measures, and magnetic resonance imaging (MRI). Each of these measures will be described, and data are reported among individuals with overweight and obesity, binge eating, and healthy weight, as well as associations with overeating and weight gain when available.

### Assessment of FCR in humans using self-report, psychophysiological measurements, or behavioral assessments

When assessed through self-reported cravings, wanting and urges to eat, FCR is typically measured on a Likert or VAS scale. Other self-report questionnaires that measure FCR concepts, include the Power of Food scale, Eating in the Absence of Hunger questionnaire, Food Cravings Questionnaire, Child Eating Behavior Questionnaire, Adult Eating Behavior Questionnaire, Reward-Based Eating Drive Scale and the Food Cue Sensitivity Questionnaire. The Power of Food scale (PFS) [82] assesses appetite for high-palatable foods, and includes three subscales; Food Available, Food Present, and Food Tasted. The Eating in the Absence of Hunger questionnaire [83, 84] assesses eating when exposed to food when physically satiated, and has three subscales; Negative Affect, External, and Fatigue/Boredom. The Food Craving Questionnaire State Version (FCQ-S) [85] assesses cravings using a multidimensional approach, and includes five subscales; an Intense Desire to Eat, Anticipation of Positive Reinforcement, Relief from Negative States, Lack of Control over Eating, and Hunger. The Child Eating Behavior Questionnaire (CEBQ) [86] includes a food responsiveness subscale that assesses overeating and desires to eat outside of typical hunger. This questionnaire has been adapted for adults [87] and babies [88]. The Reward-Based Eating Drive Scale (RED) includes questions evaluating lack of control over eating, lack of satiation, and preoccupation with food [89]. The Food Cue Reactivity Scale (FCRS) is a newly validated questionnaire that assessed uncontrolled eating and food cue rumination [90].

There are also several tasks that can be used to assess FCR, including psychophysiological tasks, attentional bias assessments as well as the eating in the absence of hunger (EAH) paradigm [91]. Psychophysiological assessments of FCR include cephalic phase responses (salivation, blood pressure, heart rate, heart rate variability among others) [92] that prepare the gastrointestinal tract for the optimal processing of food [93]. Attentional bias, or how individual’s attention is drawn toward or away from food cues, can be measured by reaction time, eye movements, or event related potentials [94]. The EAH paradigm typically includes a meal in which a child eats until physically full, and then is left alone with multiple snacks for a period of time (i.e. 10 min), and the amount of food consumed is measured. Those who eat more in the EAH paradigm could be considered to have high FCR, since they overeat when exposed to food cues when physically full. The EAH paradigm could be considered as similar to CPF in rodents.

Data show that exposure to food cues can increase cravings in both healthy individuals and those with overweight, obesity or binge eating. Research shows that exposure to real food is associated with increased self-reported cravings in individuals with overweight or obesity and those of healthy weight [95]. Interestingly, both real life and virtual reality exposure to food cues elicit cravings compared to neutral cues [96]. Among college females, food exposure was associated with changes in heart rate, heart rate variability (HRV), salivation, blood pressure, skin conductance and gastric activity, with significant correlations between blood pressure and cravings [97]. Another study showed that food craving intensity (as measured by the FCQ-S) significantly increased in individuals with binge eating and controls after watching a 5-min video clip showing food and nonfood advertisements [98].

Importantly, higher levels of FCR are associated with changes in physiology. Data shows that exposure to real food is associated with anticipatory increased heart rate, blood pressure (BP), skin response, [97, 99, 100] salivation [96], and decreased heart rate variability [95, 97, 101]. Several studies show that these food-induced physiological responses are altered in individuals with overweight, obesity, or binge-eating. For example, after viewing and smelling pizza, individuals with overweight or obesity have increased salivation and enhanced desire for food compared to those with a healthy weight [102]. Another study found that after repeated exposure to food cues, women with obesity, compared to those with healthy weight, showed delayed decline of salivation response, suggesting a reduction of extinction of the salivary response to food cues [103]. Similarly, children with obesity have greater cue-related salivation compared to children who are healthy weight, which was associated with increased food consumption [104]. Individuals with higher levels of FCR may experience increased salivation and a delay in decline of salivary response suggesting increased level and duration of arousal in response to food cues.

These findings are mirrored by the data on attentional resources. Using EEG, data show that viewing pictures of high-calorie food elicits enhanced LPP amplitudes compared with pictures of non-food and low-calorie food [105, 106]. Research shows that both women with obesity and those with a healthy weight show increased attention to food images in a fasted state, however, only women with obesity show increased attention to food images in a satiated state [107]. A recent review reports that individuals who engage in binge eating behavior exhibit an attentional bias toward food cue, in the automatic facilitated attentional engagement and purposeful attentional disengagement stages [108]. Thus, food cues capture attention, and in individuals with higher FCR, food cues may capture attention faster and there may be difficulties in disengaging their attention. This is consistent with emerging data on associations between food preoccupation and emotional eating [109, 110].

## Neural understandings of FCR in humans

**Fig. 2:**
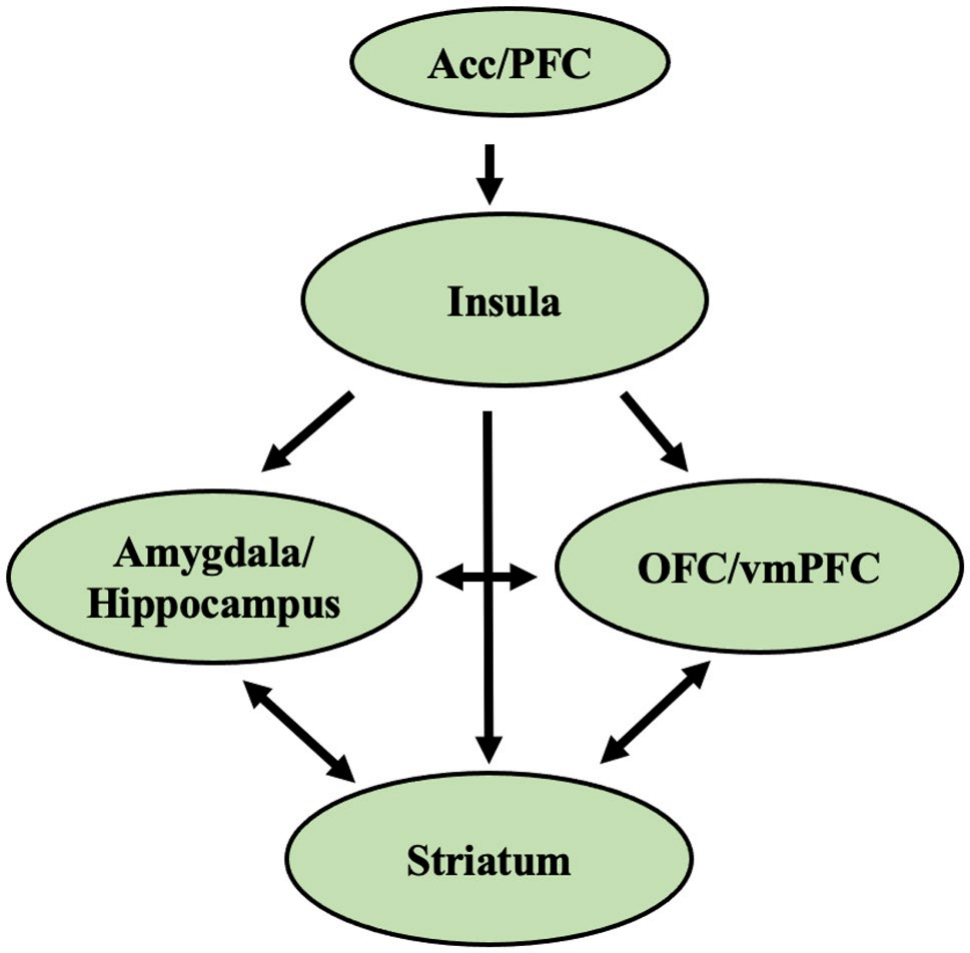
Neural regions implicated in food cue reactivity

Neural FCR can be assessed using MRI and is typically seen in brain regions associated with reward, motivation, learning, and inhibitory control systems. These fMRI paradigms use either pictures of food or tastes to measure FCR among individuals with overweight or obesity or those with healthy weight. This appetitive network includes the hippocampus [111], the amygdala [112, 113], the insula [113], the striatum, [114, 115] anterior cingulate cortex (Acc) [116], the orbitofrontal cortex (OFC) and prefrontal cortex (PFC) (see Fig. 2) [113, 117].FCR also recruits brain regions known to underlie object recognition, gustatory, and somatosensory processing like the lateral occipital gyrus, primary gustatory cortex (comprised of the anterior insula and frontal operculum), and primary somatosensory cortex, respectively [118, 119].

When evaluating FCR to pictures of food, adults with obesity compared to those with a healthy weight show increased BOLD activation in the insula, caudate, orbitofrontal cortex, amygdala, nucleus accumbens, anterior cingulate cortex, pallidum, putamen, hippocampus and prefrontal cortex [113, 116, 120–124]. However, In contrast to these findings, there is decreased brain activation in individuals with obesity compared to those with healthy weight in response to food pictures is found in the anterior cingulate, lingual and superior occipital gyri, superior frontal gyrus, precentral gyrus, cingulate gyrus, dlPFC and the temporal lobe [116, 122, 125, 126]. When evaluating FCR to tastes of food, results show that individuals with obesity, compared to those with a healthy weight, show greater activation in somatosensory (Rolandic operculum and parietal operculum), gustatory (insula and frontal operculum), and reward valuation regions (amgydala, ventramedial prefrontal cortex (vmPFC)) in response to intake of milkshake or chocolate milk versus tasteless solution [114, 127–129]. Additionally research shows that individuals with obesity, compared to those with a healthy weight, show decreased activity in the striatum in response to receipt of palatable food relative to a tasteless solution [128, 130]. Some studies also show a lack of relationship between FCR and BMI [131–135], however, these mixed results may be due to mixed stimuli (pictures and tastes), small sample sizes, control conditions, and methods of analyses.

More recently, the hippocampus is being recognized as an important substrate in appetitive control (also summarized above) [136]. A growing body of research highlights the importance of hippocampal-dependent learning mechanisms in integrating external food cues with the internal/interoceptive experience which can ultimately influence FCR [49]. In humans, inflammation and reductions in gray matter in the hippocampus are associated with having obesity [137, 138]. Both adults and children with obesity show smaller hippocampal volumes, relative to those with healthy weight [137, 139, 140]. A large study among adolescents across the weight spectrum showed that BMI was not associated with hippocampal volume but was associated with measures of tissue integrity [141].

Neural responding to food cues is consistently associated with eating behavior and weight change [135]. Exposure to pictures of food and changes in the appetitive network are associated with preference for high calorie foods, changes in caloric intake [142, 143] and weight gain [144, 145]. Responses to chocolate cues in the dorsal striatum predicted later chocolate consumption among a group of participants who were exposed to chocolate as part of a “taste test” prior to the scan, compared to a control group [146]. Similarly, activity in the medial OFC, amygdala, insula, and nucleus accumbens while viewing high-calorie foods predicted higher-fat food choices after an fMRI scan [142]. In one study, midbrain and medial OFC activity related to milkshake tastes during an fMRI scan positively predicted later ad libitum milkshake consumption among adolescents [147]. Another found that variability in nucleus accumbens activity to milkshake consumption was related to dietary disinhibition and variability in ad libitum food intake [148, 149]. FCR in the nucleus accumbens, significantly predicted strength of food desires, enactment of those desires, and the amount eaten [150]. In children, activation in the hippocampus was associated with increased in the eating in the absence of hunger paradigm [139]. Higher activity in the nucleus accumbens in response to food pictures predicts weight change over 6-months [144]. A more recent study showed that increases in the motor processing areas, but not in the striatum, predicts BMI gain over 3 years [151]. Finally, a growing body of work focuses on identifying individual patterns of brain activity that predict weight change [13, 144, 152]. In summary, these studies point to a strong association between widespread neural activation, overeating and obesity risk, confirming that neural FCR is an important factor in weight gain in humans.

As discussed in the preclinical studies, the appetite-promoting hormone, ghrelin, plays an important role in FCR and can influence neurogenesis in the hippocampus. While leptin is also considered a hormone that influences appetite (in an opposite direction as ghrelin), ghrelin seems to activate areas associated with visual processing and attention while leptin is associated with activation of areas associated with anticipation of higher levels of reward [153]. Specifically, higher circulating levels of ghrelin are associated with activity in neural areas associated with visual processing (middle occipital gyrus, fusiform gyrus), reward (caudate) and the limbic system (amygdala, thalamus) [154, 155], and reduction in ghrelin levels is associated with dorsolateral prefrontal cortex activation to food cues and reduction in craving ratings for food [156]. Among individuals with healthy weight, both fasting and subcutaneously injected ghrelin in a fed state increases hippocampus activation in response to pictures of high and low calorie foods, and orbitofrontal cortex activation in response to high calorie foods [153]. Interestingly, ghrelin and leptin are not associated with increased neural activity in response to food cues in the fed state [153]. A food-cue reactivity study in humans revealed that fasting ghrelin concentrations were associated with the hedonic effects of food pictures and with enhanced subjective craving when confronted with reward cues [154]. In summary, results show that similar to the preclinical work, ghrelin seems to play a significant role in FCR in humans.

### Cue reward learning

As mentioned earlier, FCR is dependent on learning the relationship between a “cue” and food. Initially, the food elicits responding directly, but over time, the responding shifts from the food to the cue predicting food. Theorists suggest that this shift during cue**-**reward learning acts to update knowledge regarding the predictive cues or attribute reward value to the cues which guides behavior [157–159] and induces motivational states (e.g. salivation, cravings, expectations to eat) that can oppose the existing physiological drive. Analogous to the US-specific PIT described above, the drive in these circumstances is selective and specific and as such, is similar to induction of appetite, or even craving, rather than induction of a more general state of hunger [160]. Initial studies evaluated food cue reward learning among humans pairing fractal images with a taste of glucose, tasteless saliva or no cue among healthy adult volunteers [161, 162]. These studies demonstrated learning as predicted, and there was a shift in the peak of the hemodynamic curve in the ventral striatum and orbitofrontal cortex from the taste itself to the cue that predicted the taste.

A behavioral study evaluated Pavlovian learning to innocuous cues associated with a hedonic and non-hedonic stimulus among young adults with overweight or obesity and those with healthy weight [163]. The conditioning paradigm presented innocuous visual cues (square, triangle) on a computer screen which were associated with a taste of chocolate milk or water, and swallowing frequency was measured by EMG recordings as a non-invasive estimate of salivation [164] for two minutes at baseline and after the acquisition trials [164]. Results showed a significant difference between chocolate and water swallowing at acquisition compared to baseline for individuals with obesity. Conversely, for healthy weight participants, there was no significant difference between chocolate and water swallowing at acquisition compared to baseline. These results suggest that participants with overweight or obesity learned the relationship between innocuous cues and hedonic vs. non-hedonic liquids faster than lean participants.

To our knowledge there have only been two published fMRI studies to date that link Pavlovian cue reward learning to weight, and both have used different stimuli and methods. The first study evaluated 35 adolescent girls who viewed cues (diamond, square, circle) that predicted a taste of milkshake or tasteless solution in the MRI [165]. Results showed that individual slopes of cue-reward learning in the ventral pallidum were significantly associated with BMI over a 2-year follow-up. The second study among 153 adolescents used real life cues (glasses of milkshake and water) that signaled impending taste of milkshake or tasteless solution [166]. Results showed increased BOLD activation in the orbitofrontal cortex predicted future body fat gain over three years, but not BMI change. Lower BOLD activity to the cue contrast in the bilateral superior visual cortex, lingual gyrus, and ventromedial prefrontal cortex also predicted body fat gain over three years. Since this study used pictures of glasses of milkshake and water as cues, the participants already had associations with the outcome from other learning experiences, and thus this last study did not purely test cue-reward learning. Cue-reward learning could be another individual difference that could be used to identify individuals at high risk for increased FCR.

### Conclusions

FCR can be measured using several different methods in humans, including self-report, questionnaires, psychophysiological measures, and MRI. Emerging research demonstrates the relationship between FCR, eating and weight. Food pictures and tastes activate the appetitive network, which includes the hippocampus, amygdala, insula, striatum, anterior cingulate cortex, orbitofrontal cortex and prefrontal cortex. Emerging research suggests that ghrelin is an important hormone linked to attention and visual processing contributing to FCR which can also impact hippocampal neurogenesis. Finally, food cue-reward learning seems to be implicated in overeating and obesity, however understanding which individuals may be at risk for increased food cue-reward learning and how to intervene has yet to be elucidated.

### Species parallels

Comparisons between the preclinical and human study literature reviewed above identify several parallels in FCR underlying mechanisms. At the behavioral level, FCR is reliable and robust in both humans and rodents and can be triggered by both primary food cues (cues directly associated with food, e.g., food pictures, odors) and cues that are associated with palatable food via Pavlovian conditioning (e.g., fast food logos, otherwise neutral discrete lights and tones). In both species, such cues can not only stimulate elevated food consumption, but also increase appetitive operant responses that are conditioned to lead to palatable food access. FCR responses, both biological and behavioral, are present in individuals with healthy weight and lean rodents, but are heightened in humans with overweight, obesity, or binge eating, as well as in rodents that are either obese or particularly susceptible to obesity development. At the neuronal level, several common brain regions have been associated with FCR in both animal models and human studies, including the nucleus accumbens, the amygdala, the orbitofrontal cortex, and the hippocampus. Finally, the orexigenic stomach-derived hormone ghrelin is linked with elevated FCR in both humans and rats, as both species increase physiological ghrelin release in response to food-associated cues, show increased behavioral FCR with either physiological or pharmacological increases in ghrelin signaling, and show functional connections between FCR and ghrelin action in the hippocampus. That such strong behavioral, neural, and endocrine parallels exist between FCR preclinical and human studies is encouraging in the sense that mechanistic rodent models may lead to scientific advances in curbing FCR that will be relevant for human obesity prevention and treatment.
